# Supplementary material for: Using UK Biobank data to establish population-specific atlases from whole body MRI
Source: Commun Med (Lond). 2024 Nov 19;4:237. doi: 10.1038/s43856-024-00670-0 (PMC11577111; doi:10.1038/s43856-024-00670-0)
Supplement: Supplementary file 2 — Supplementary Information [file 43856_2024_670_MOESM2_ESM.pdf]

Supplementary Information

Supplementary Table 1. Summary of the Affine and Deformable Registration hyperparameters.

Method	Software	Diss. Metric	Levels	Transformation	Spacing	Reg.	Optimizer	Learning Rate
**Affine**	Deepali[2]	NCC	3	-	-	-	Adam	10^{-3}
**Deformable**	Deepali[2]	LNCC	3	FFD[1]	16 pixels	0.1	Adam	10^{-3}

We report the registration tool, the dissimilarity metric, resolution levels, transformation type, control point spacing, regularisation, optimizer and associated learning rate.

Supplementary Figure 1. Overview of the atlas unbiasing for all groups.

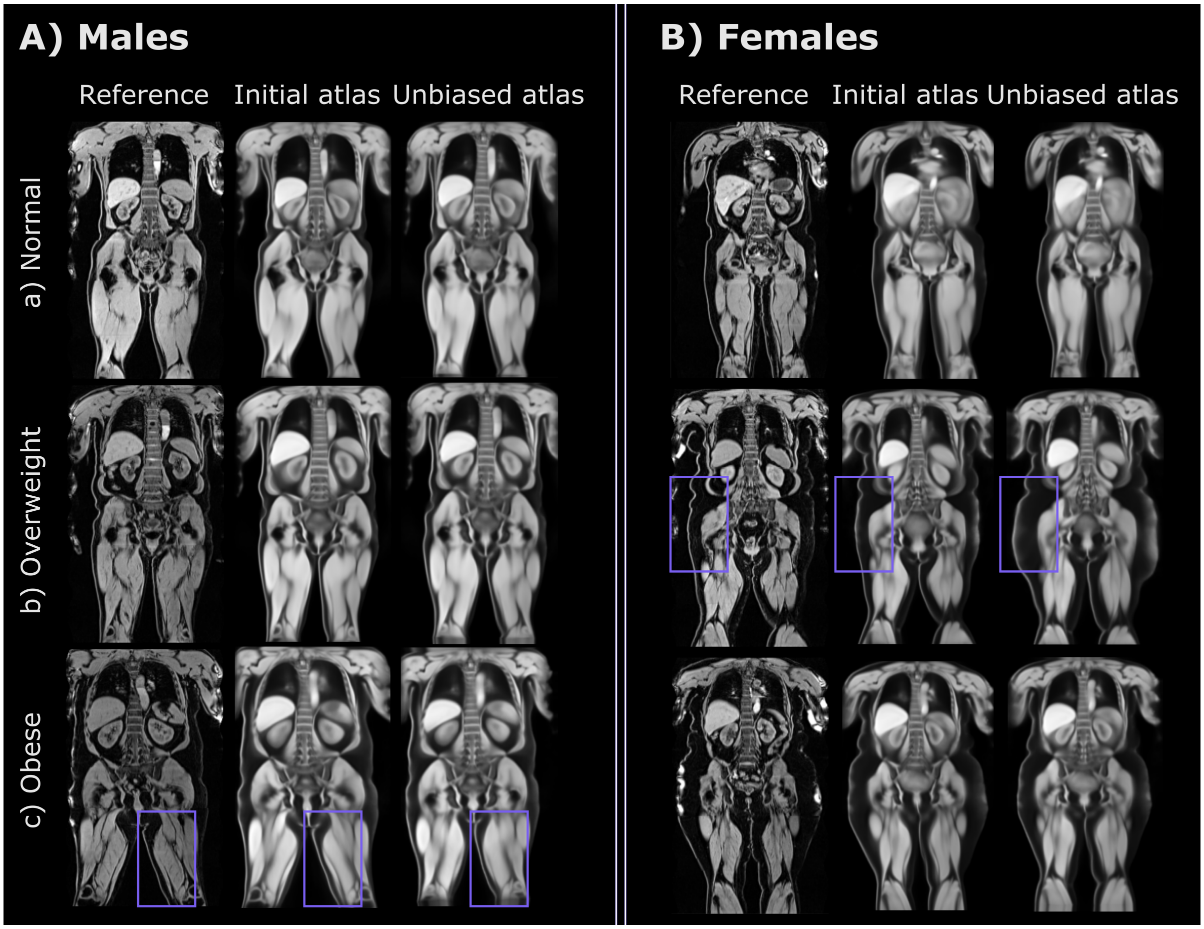

Overview of the unbiasing for the female and male atlases for the normal weight (a), overweight (b), and obese (c) groups. The purple bounding boxes highlight regions where the unbiasing deformations are visible.

Supplementary Figure 2. Overview of the normal weight atlases.

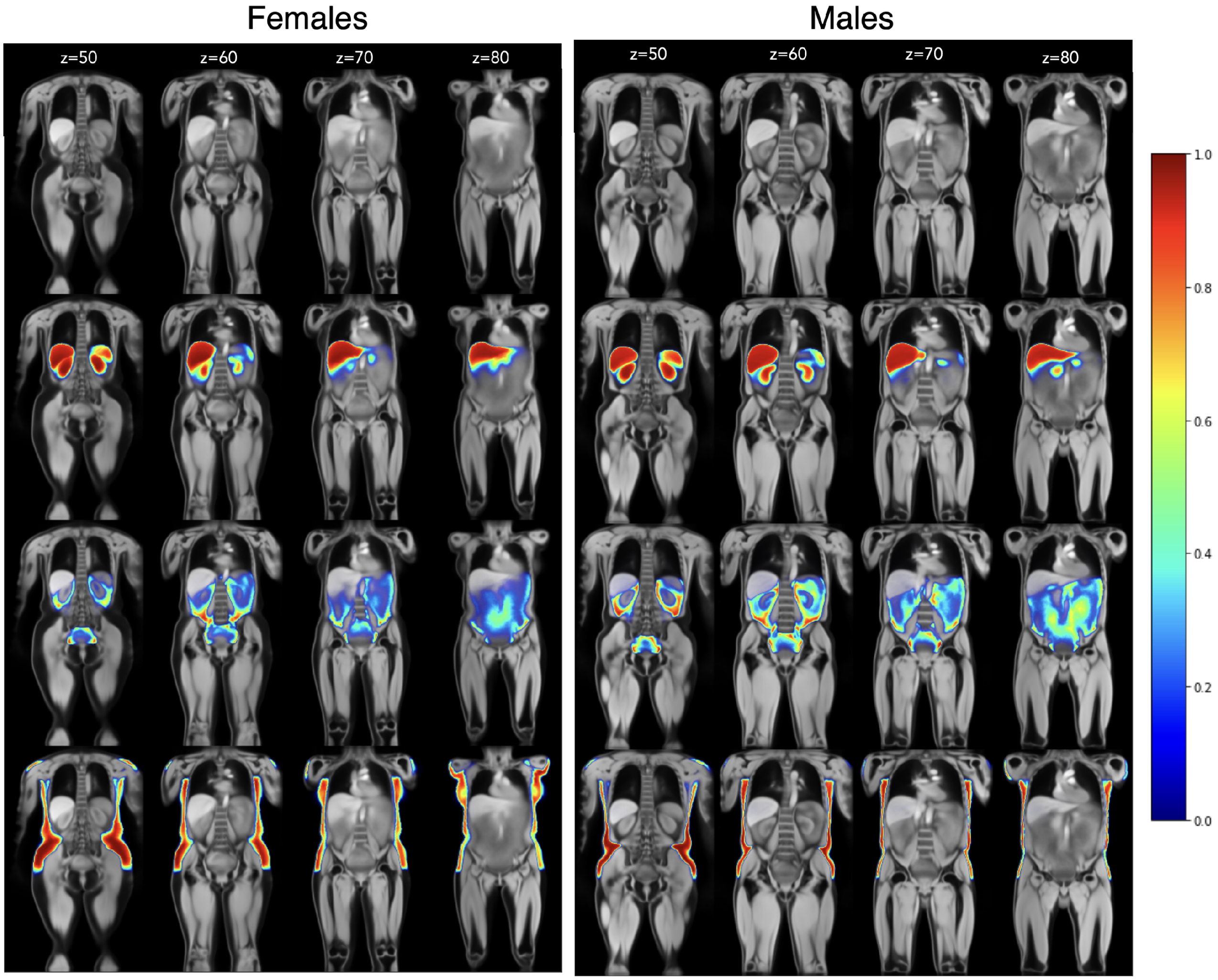

Overview of the **normal weight** atlas across multiple slices for females and males. The water-contrast anatomical atlas is shown alongside the abdominal organs label atlas, the visceral fat label atlas, and the subcutaneous fat label atlas. Each column shows a different slice, and each row shows a different label for the same atlas, and the colour bar represents the probability of the labels.

Supplementary Figure 3. Overview of the overweight atlases.

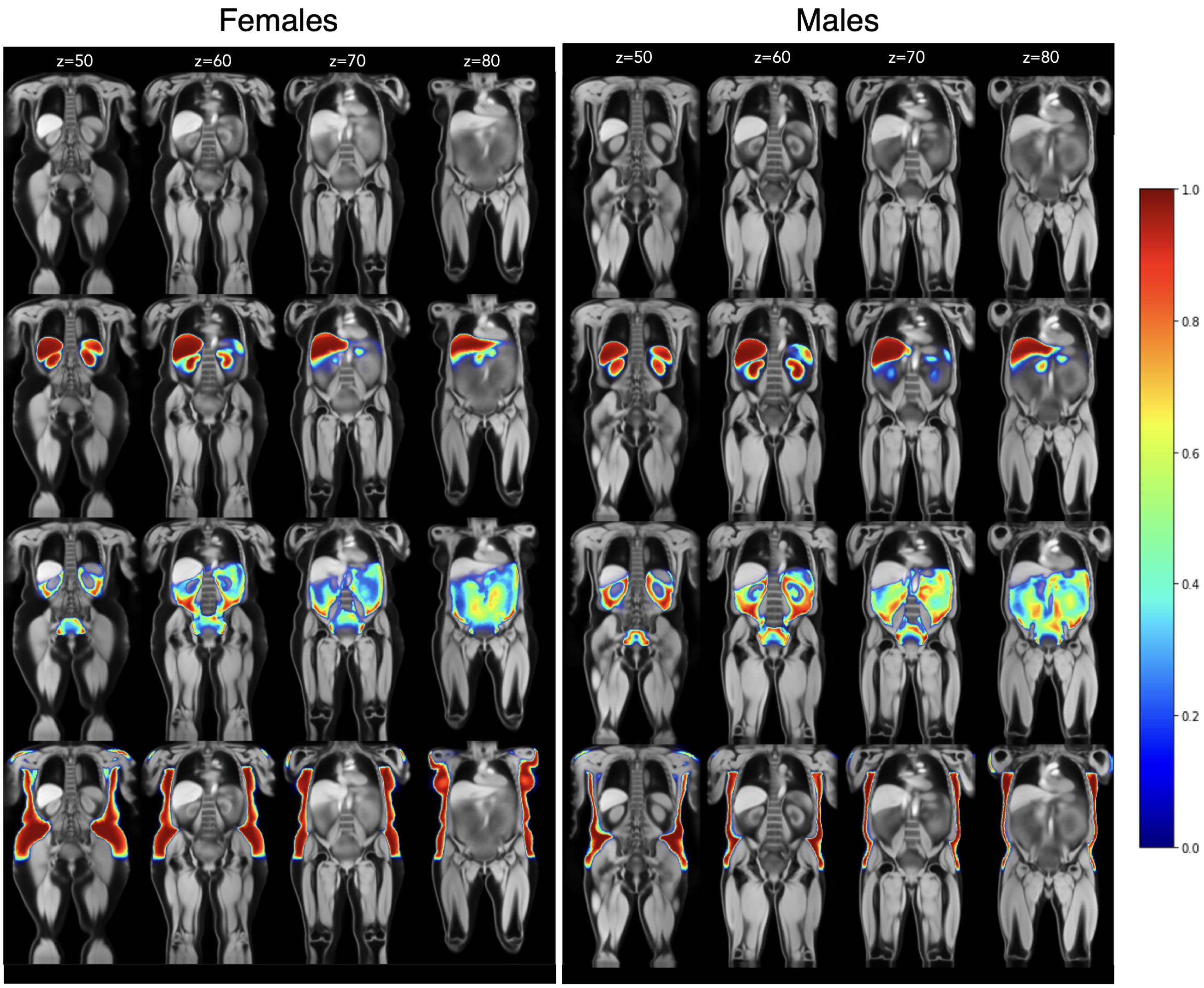

Overview of the **overweight** atlas across multiple slices for females and males. The water-contrast anatomical atlas is shown alongside the abdominal organs label atlas, the visceral fat label atlas, and the subcutaneous fat label atlas. Each column shows a different slice, and each row shows a different label for the same atlas, and the colour bar represents the probability of the labels.

Supplementary Figure 4. Overview of the obese atlases.

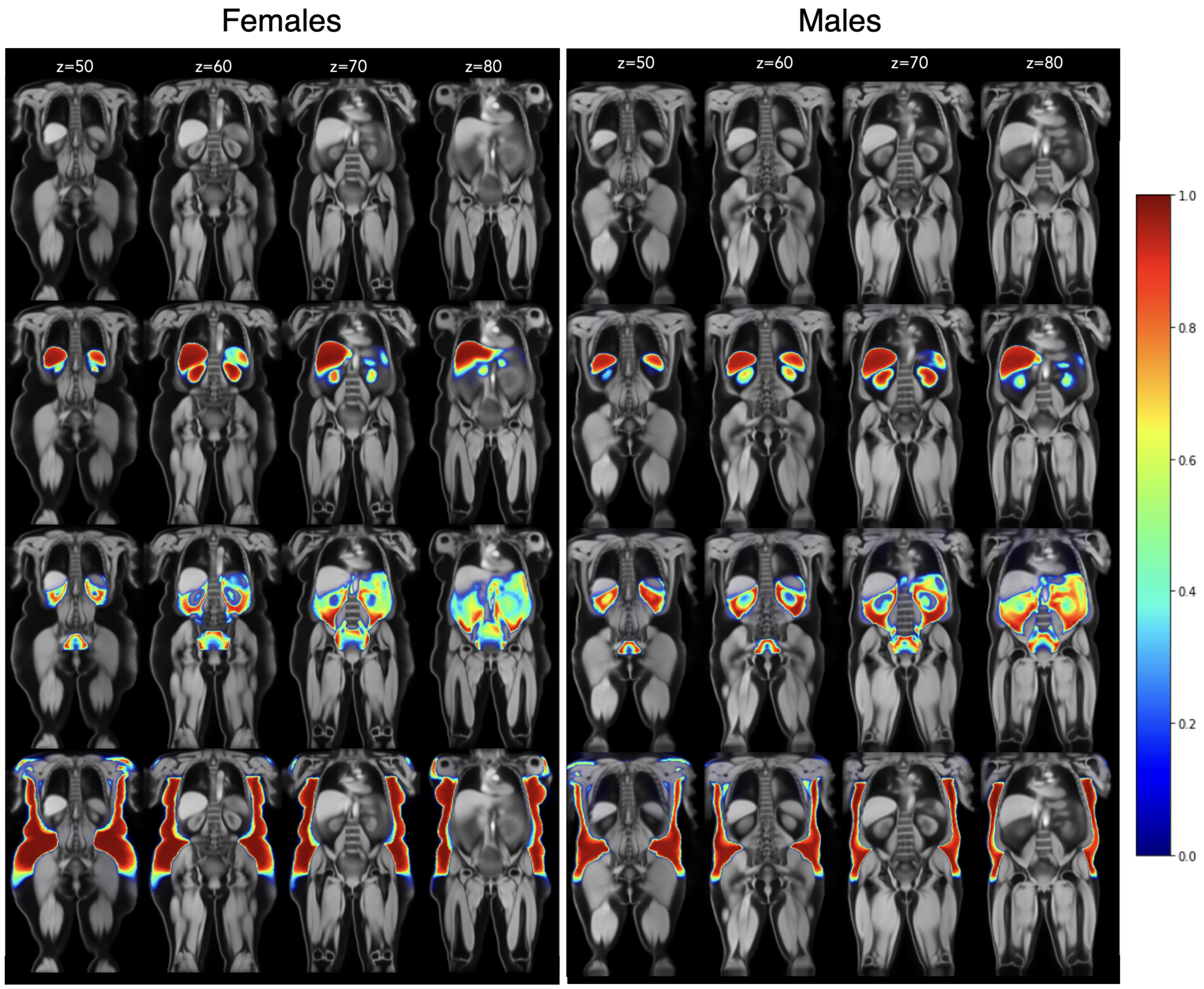

Overview of the **obese** atlas across multiple slices for females and males. The water-contrast anatomical atlas is shown alongside the abdominal organs label atlas, the visceral fat label atlas, and the subcutaneous fat label atlas. Each column shows a different slice, and each row shows a different label for the same atlas, and the colour bar represents the probability of the labels.

Supplementary Figure 5. Overview of the VBM results for females with CAD.

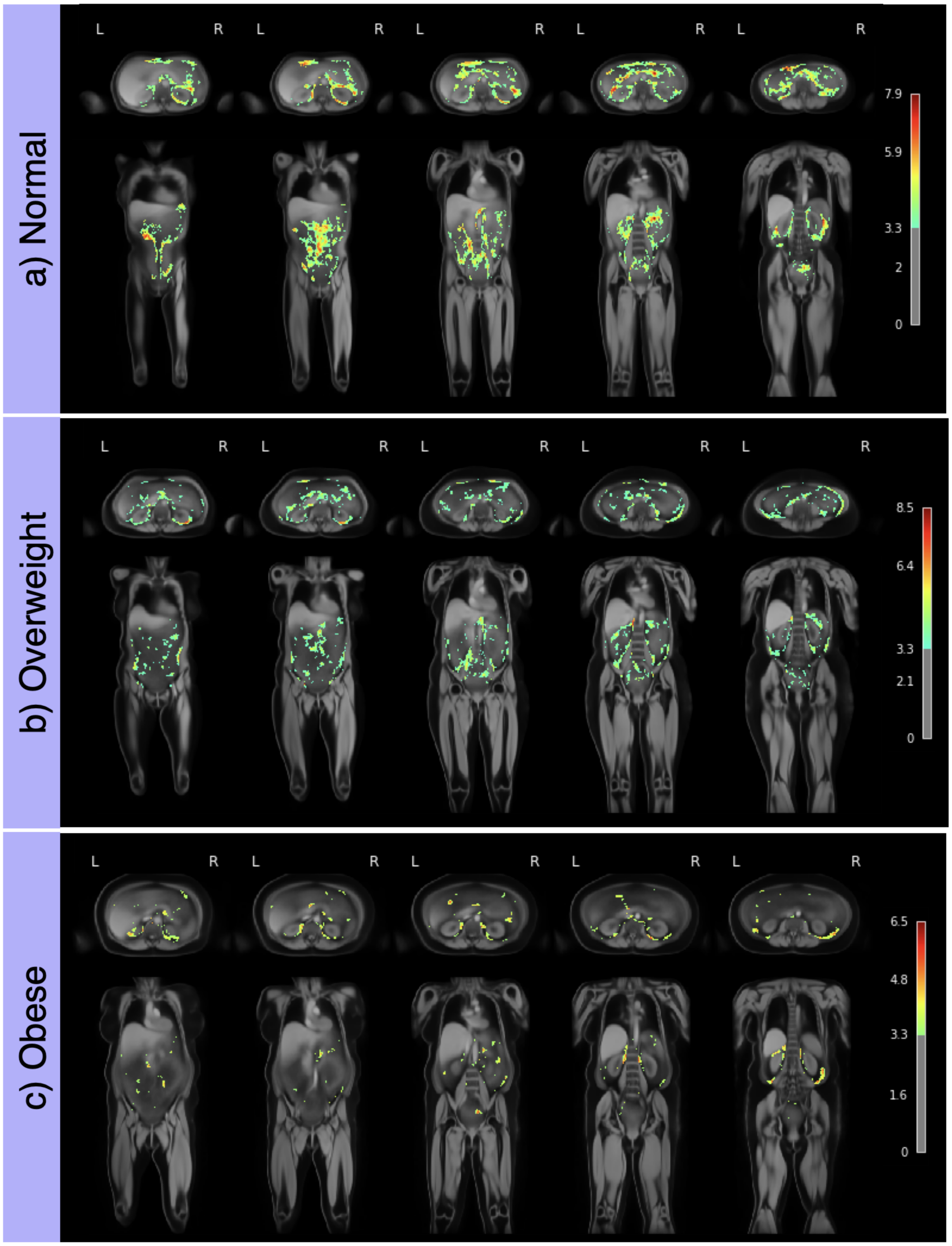

Overview of the VBM results for **females** with **CAD**. The significant voxels ($p < 0.001$) in the visceral fatty tissue for patients with coronary artery disease for three BMI groups: normal ($n = 363$), overweight ($n = 425$), and obese ($n = 312$). The columns show different slices for both axial and coronal views. The colour bars on the right describe the z-score; higher values indicate significant differences in visceral fat between the groups.

Supplementary Figure 6. Overview of the VBM results for males with CAD.

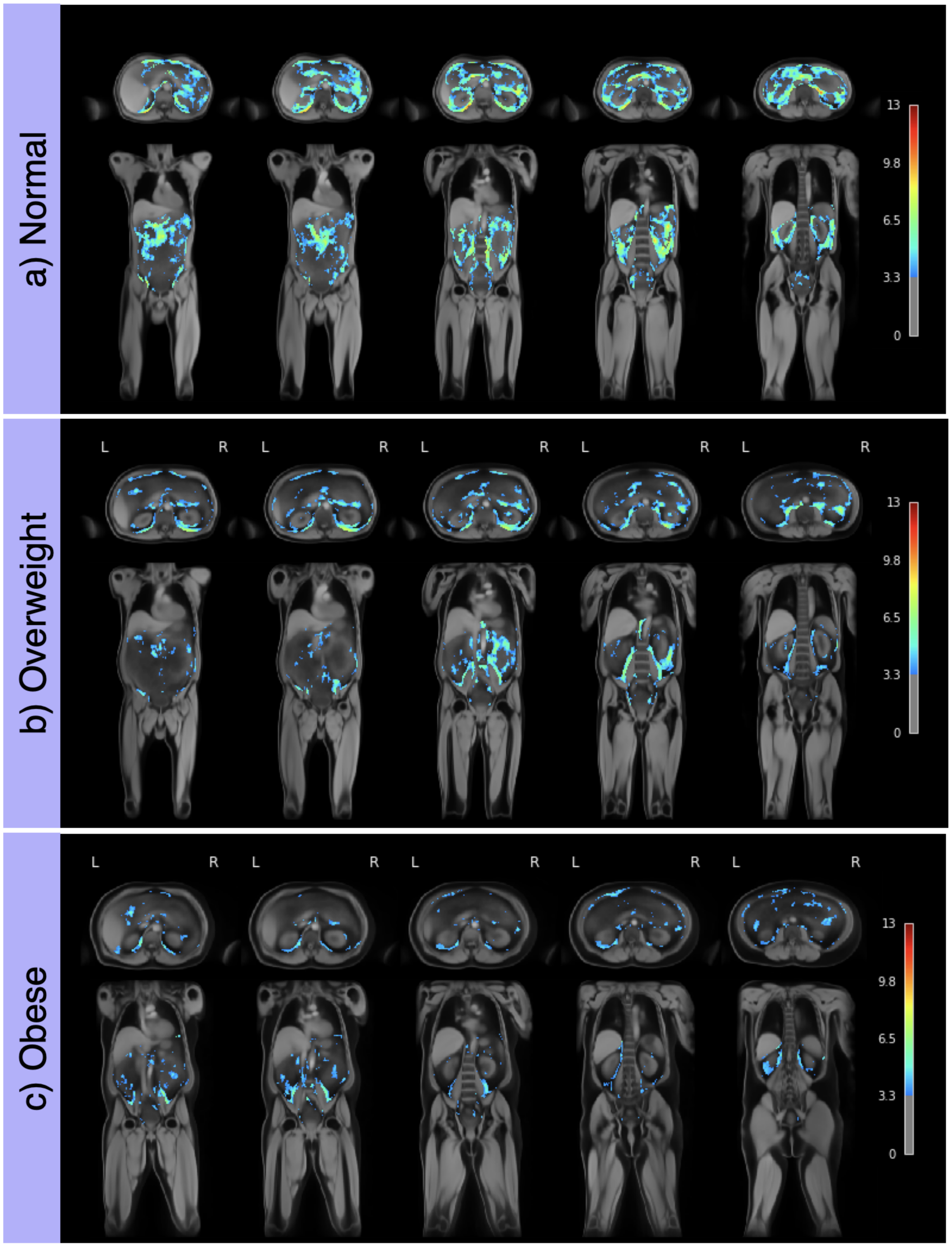

Overview of the VBM results for **males** with **CAD**. The significant voxels ($p < 0.001$) in the visceral fatty tissue for patients with coronary artery disease for three BMI groups: normal ($n = 700$), overweight ($n = 800$), and obese ($n = 705$). The columns show different slices for both axial and coronal views. The colour bars on the right describe the z-score; higher values indicate significant differences in visceral fat between the groups.

Supplementary Figure 7. Overview of the VBM results for females with diabetes.

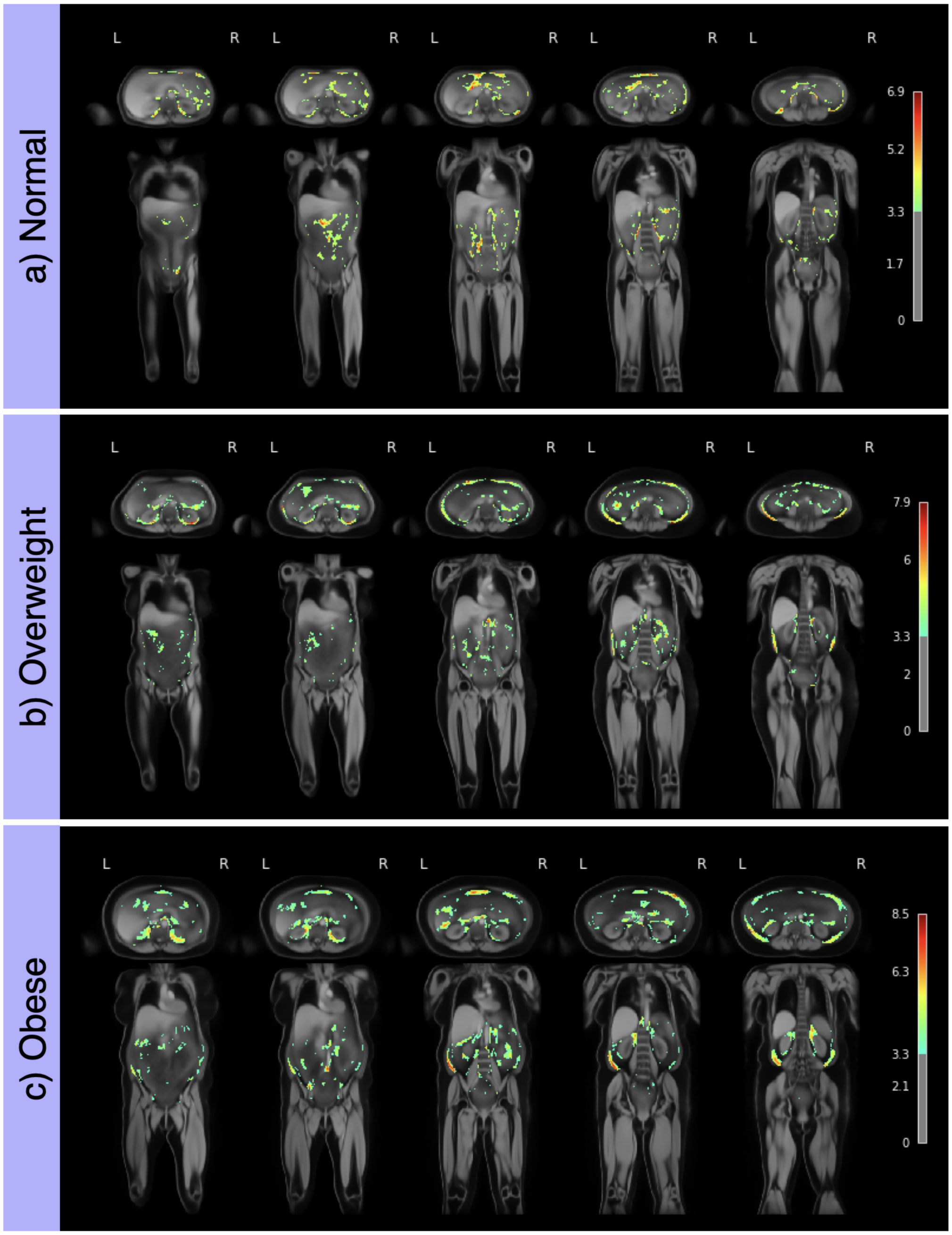

Overview of the VBM results for **females** with **diabetes**. The significant voxels ($p < 0.001$) in the visceral fatty tissue for patients with type 2 diabetes for three BMI groups: normal ($n = 121$), overweight ($n = 267$), and obese ($n = 393$). The columns show different slices for both axial and coronal views. The colour bars on the right describe the z-score; higher values indicate significant differences in visceral fat between the groups.

Supplementary Figure 8. Overview of the VBM results for males with diabetes.

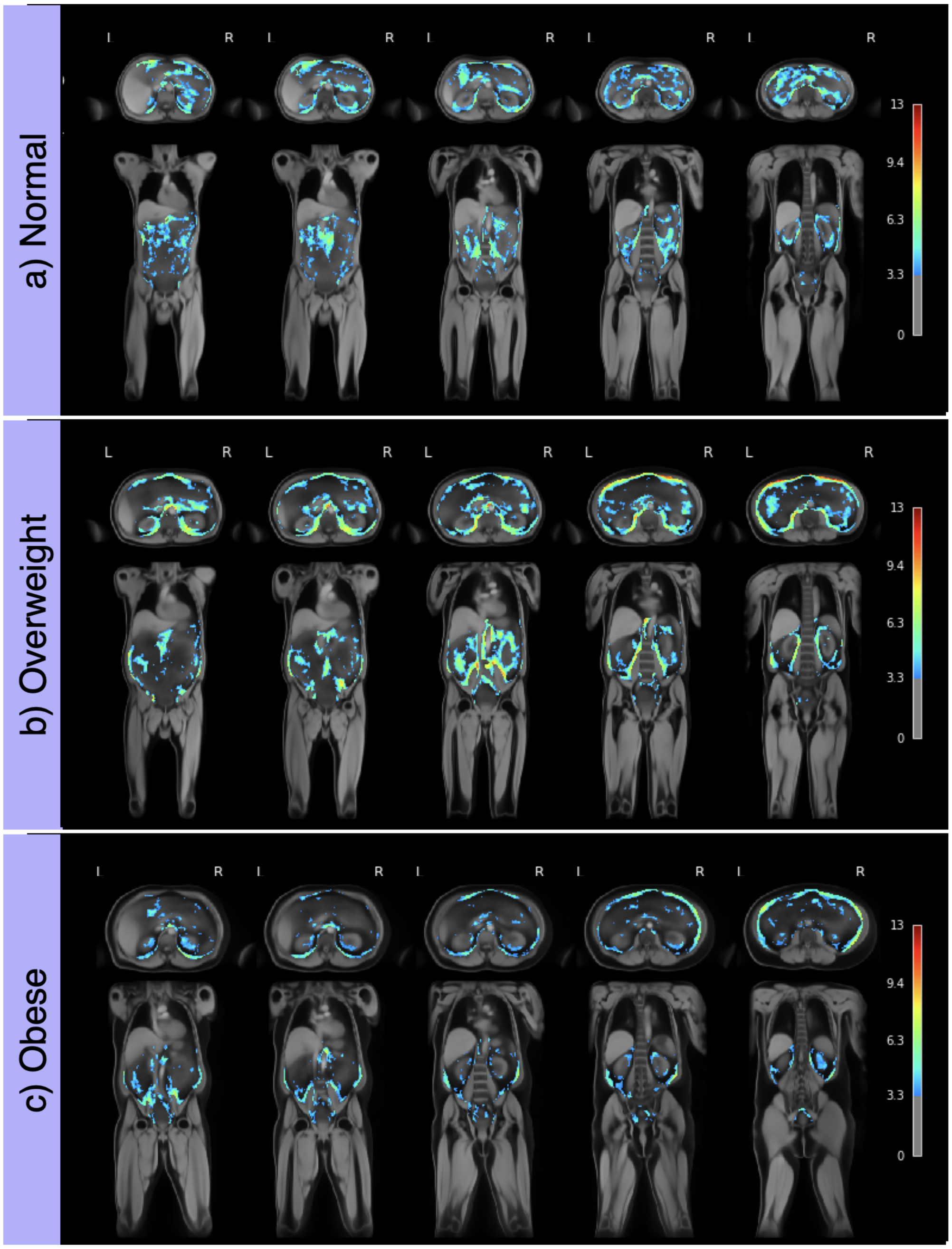

Overview of the VBM results for **males** with **diabetes**. The significant voxels ($p < 0.001$) in the visceral fatty tissue for patients with type 2 diabetes for three BMI groups: normal ($n = 220$), overweight ($n = 647$), and obese ($n = 605$). The columns show different slices for both axial and coronal views. The colour bars on the right describe the z-score; higher values indicate significant differences in visceral fat between the groups.

References

1. Marc Modat, Pankaj Daga, Manuel Jorge Cardoso, Sébastien Ourselin, Gerard R. Ridgway, and John Ashburner. Parametric non-rigid registration using a stationary velocity field. *2012 IEEE Workshop on Mathematical Methods in Biomedical Image Analysis*, pages 145–150, 2012.
2. Andreas Schuh, Huaqi Qiu, and HeartFlow Research. deepali: Image, point set, and surface registration in PyTorch.
